# Fixed Effects Modelling for Provider Mortality Outcomes: Analysis of the Australia and New Zealand Intensive Care Society (ANZICS) Adult Patient Data-Base

**DOI:** 10.1371/journal.pone.0102297

**Published:** 2014-07-16

**Authors:** John L. Moran, Patricia J. Solomon, for the ANZICS Centre for Outcome and Resource Evaluation (CORE) of the Australian and New Zealand Intensive Care Society (ANZICS)

**Affiliations:** 1 Department of Intensive Care Medicine, The Queen Elizabeth Hospital, Woodville, South Australia, Australia; 2 School of Mathematical Sciences, University of Adelaide, Adelaide, South Australia, Australia; 3 Australian and New Zealand Intensive Care Society, Carlton, Victoria, Australia; Utrecht University, Netherlands

## Abstract

**Background:**

Risk adjusted mortality for intensive care units (ICU) is usually estimated via logistic regression. Random effects (RE) or hierarchical models have been advocated to estimate provider risk-adjusted mortality on the basis that standard estimators increase false outlier classification. The utility of fixed effects (FE) estimators (separate ICU-specific intercepts) has not been fully explored.

**Methods:**

Using a cohort from the Australian and New Zealand Intensive Care Society Adult Patient Database, 2009–2010, the model fit of different logistic estimators (FE, random-intercept and random-coefficient) was characterised: Bayesian Information Criterion (BIC; lower values better), receiver-operator characteristic curve area (AUC) and Hosmer-Lemeshow (H-L) statistic. ICU standardised hospital mortality ratios (SMR) and 95%CI were compared between models. ICU site performance (FE), relative to the grand observation-weighted mean (GO-WM) on odds ratio (OR), risk ratio (RR) and probability scales were assessed using model-based average marginal effects (AME).

**Results:**

The data set consisted of 145355 patients in 128 ICUs, years 2009 (47.5%) & 2010 (52.5%), with mean(SD) age 60.9(18.8) years, 56% male and ICU and hospital mortalities of 7.0% and 10.9% respectively. The FE model had a BIC = 64058, AUC = 0.90 and an H-L statistic *P*-value = 0.22. The best-fitting random-intercept model had a BIC = 64457, AUC = 0.90 and H-L statistic *P*-value = 0.32 and random-coefficient model, BIC = 64556, AUC = 0.90 and H-L statistic *P*-value = 0.28. Across ICUs and over years no outliers (SMR 95% CI excluding null-value = 1) were identified and no model difference in SMR spread or 95%CI span was demonstrated. Using AME (OR and RR scale), ICU site-specific estimates diverged from the GO-WM, and the effect spread decreased over calendar years. On the probability scale, a majority of ICUs demonstrated calendar year decrease, but in the for-profit sector, this trend was reversed.

**Conclusions:**

The FE estimator had model advantage compared with conventional RE models. Using AME, between and over-year ICU site-effects were easily characterised.

## Introduction

Risk-adjusted mortality has been used to characterise the performance of health care providers for a number of years [1] and has generated a substantial [2] if not controversial [3] literature. Inference regarding risk-adjusted mortality is dependent on both the illness severity measure [4], [5] and the estimation method [6], [7]. Mortality probability estimation usually proceeds via conventional logistic regression [8] but a call for “Improving the statistical approach to health care provider profiling”, in particular the use of Bayesian methods, was made some 15 years ago [9]. Advances in standard statistical software packages have made such approaches feasible and a random effects or hierarchical approach to estimation, both Bayesian and frequentist, has recently been advocated [10], [11] and implemented [12].

However, such recommendation must also address certain cautions recently advanced regarding the latter methods [13], [14], in particular the reduction of variation of hospital performance by the shrinkage effect of conventional random effects models. In a wide-ranging discussion Ash and co-workers (The COPSS [Committee of Presidents of Statistical Societies]-CMS [Centers for Medicare and Medicaid Services] White Paper Committee, [15]) noted that in the presence of sufficient stand-alone hospital data and an appropriately specified model, a fixed effects approach (in this case, separate hospital-specific intercepts) would ensure “…successful adjustment for potential confounding [15].” Such endorsement has been reiterated by the empirical demonstration of the efficacy of such a fixed effects approach [6], [16]–[20]. This being said, the interpretation of β coefficients (log-odds ratios or odds ratios) from such a fixed effects model as “substantive effects” may be problematic due to unobserved heterogeneity and confounding of effects [21]. Furthermore, as argued by Angrist, structural parameters (that is, the β coefficients) may be of theoretical interest, but must be “…converted into causal effects if they are to be of use for policy evaluation or determining whether a trend association is causal” [22].

The Australian and New Zealand Intensive Care Society (ANZICS) adult patient data base (APD) [23], administered by the Centre for Outcome and Resource Evaluation (ANZICS CORE) [24], is a high-quality bi-national intensive care patient data-base, and satisfying the above data requirements, would be entirely suited to such a modelling approach. Using recent data from this data-base (calendar years 2009–2010), the purpose of this paper was to (i) develop a predictive fixed effects logistic model, enumerate its properties and compare these with conventional random effects models and (ii) characterise the relative performance of ICUs (with respect to mortality outcomes), using the fixed effects model, on the probability and other scales using average marginal effects (AME) [21], [25], [26] or “marginal standardisation” [27], adjusting for the multiple comparisons so undertaken [28].

## Methods

### Ethics statement

Access to the data was granted by the ANZICS Database Management Committee in accordance with standing protocols; local hospital (The Queen Elizabeth Hospital) Ethics of Research Committee approval was waived. The data set analysed is the property of the ANZICS Data base and contributing ICUs and is not in the public domain. The data are available to personnel of the ANZICS Data base and contributing ICUs under specific conditions and upon written request.

### Data management

As previously described [29], [30] the ANZICS APD was interrogated to define an appropriate patient set over the time period 2009–2010. In brief, physiological variables collected in accordance with the requirements of the APACHE III algorithm [31] were the worst in the first 24 hours after ICU admission, and all first ICU admissions to a particular hospital for the period 2009–2010 were selected. Records were used only when all three components of the Glasgow Coma Score (GCS) were provided; records for which all physiologic variables were missing were excluded, and for the remaining records, missing variables were replaced with the normal range and weighted accordingly [32]. Ventilation status in the data base was recorded with respect to invasive mechanical ventilation on or within the first 24 hours of ICU-admission. Exclusions: unknown hospital outcome; patients with an ICU length of stay  = 4 hours, and patients aged <16 years of age. Continuous variables (age, APACHE III score and annual-volume) were centred for model stability considerations. Categorical predictors were parameterized as indicator variables with the reference level ( = 0) indicated in parentheses in the following list: year (2009); gender (female); ventilation (non-ventilated); ICU-level, as defined in the ANZICS data dictionary [33], as Rural/Regional, Metropolitan, Tertiary and Private (Tertiary); geographical-location, that is New Zealand and the States of the Commonwealth of Australia (New South Wales (NSW), the largest contributor); ICU source, that is, patient transfer from another hospital (no transfer); patient surgical status as post-elective surgery, post-emergency surgery and non-surgical (non-surgical); descriptors of ICU admission primary organ system dysfunction, these being a consolidation of the diagnostic categories of the Acute Physiology and Chronic Health valuation (APACHE) III algorithm: cardiovascular, gastrointestinal, metabolic, neurologic, respiratory, trauma, renal/genitourinary (cardiovascular); ICU site (first site of the sequential numeric ordering of ICUs). Annual (“annualised” [34]) volume, determined for each ICU recorded in the database, was also considered as a (decile) categorical variable (first decile) [30]; see below.

### Statistical analysis

Analyses were performed using Stata (Version 13, 2013; College Station, TX); continuous variables were reported as mean (SD), except where otherwise indicated, and statistical significance was ascribed at P≤0.05.

Three separate models were estimated:

- logistic regression: for patient *i* in provider *k* the logit (log-odds) of hospital mortality probability (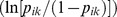) was given as: 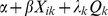, where 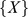 was a set of independent predictor variables and 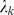 represented the additional risk effect of the *k*th provider (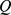); that is, provider effects were fixed [6], [35]. Appropriate accounting of patients' within ICUs was obtained using the robust cluster variance option [36] of Stata; given as 
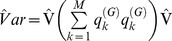
, where 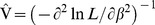 is the conventional estimator of variance, 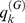 is the contribution of the *k*th provider to 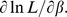 Assuming an additive likelihood function: 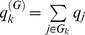; 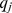 being a row vector of observations [37], [38].
- random effects (or empirical Bayes) models, random intercept and random coefficient, as 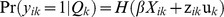 for *k* = 1,…,*Q* providers (provider *k* consisting of *i* = 1,…,n observations). The 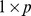 row vector 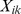 were the covariates for fixed effects and 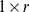
vector 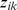 the covariates corresponding to the random effects (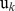) and were used to represent both random intercepts and random coefficients. 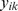 was the binary (0/1) outcome variable (hospital mortality) and *H* the logistic cumulative distribution function.
  - In the random intercept model, 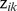 was a scalar 1.
  - In the random coefficient (“slope”) model, the centred APACHE III score (as a dominant predictor of hospital mortality [29]) was used; an unstructured covariance matrix was implemented (that is, the usual (symmetric) variance-covariance matrix which includes components of covariance between the random effects).
  - Model estimation used (7-point) adaptive quadrature, a computational method used to approximate the marginal likelihood by numerical integration [39]; the modelling perspective was frequentist.

Seasonality of mortality was addressed using trigonometric (sine and cosine) terms for yearly, 6 monthly and weekly effects after Stolwijk [40].

For fixed model variables, detailed above in “Methods”, sets of parameter coefficients were tested using a global Wald test [41] and model development and comparison was guided by the Akaike Information Criterion (AIC), with the Bayesian Information Criterion (BIC) for non-nested models (28). In the presence of specific (fixed) ICU effects (parameterised as a multilevel (indicator) categorical variable), in the FE model only, particular attention was directed to the identification of variable collinearity with other model fixed effects variables, using the Stata module “_rmcoll” [42]. Model adequacy was gauged by the traditional criteria of discrimination (receiver operator characteristic curve area, AUC) and calibration (Hosmer-Lemeshow (H-L) statistic); albeit the H-L statistic will invariably be significant (P<0.1 and H-L statistic >15.99) in the presence of a large N [43] and increments to the grouping number (default  = 10) of the H-L test were appropriately made [44]. Model residual analysis was undertaken using (i) distributional diagnostic plots, specifically the comparison of the empirical distribution of the residuals against the normal distribution; Q-Q and P-P plots [45]) and (ii) the “binned residual” approach (initially presented for small samples) as recommended by Gelman and Hill [46]: the data were divided into categories (bins) based upon the fitted values and the average residual (observed minus expected value) versus the average fitted value was plotted for each bin; the boundary lines, computed as 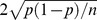 where *n* was the number of points per bin, indicated ± 2SE bounds, within which one would expect about 95% of the binned residuals to fall.

Confidence intervals (CI) of the ICU standardised mortality ratio (SMR) were calculated by back-transformation from the variance of the (log) observed / predicted mortality using the Taylor series approximation [47]. The multivariate relationships (joint distribution) between various estimates were displayed using biplots [48]. Biplots consist of lines, reflecting the dataset variables, and “dots” to show the observations. The length of the lines approximates the variances of the variables (the longer the line, the higher is the variance) and the cosine of the angle between the lines approximates the correlation; the closer the angle is to 90, or 270 degrees, the smaller the correlation (orthogonality or un-correlated); an angle of 0 or 180 degrees reflecting a correlation of 1 or −1, respectively [49].

Exploration of comparative ICU site performance, by ICU level and calendar year, relative to the grand observation-weighted mean [15], [19] on both the predictive probability (the default), (log) odds ratio (OR) and risk ratio (RR) scales was undertaken using the “margins” and “contrast” operators of Stata, with the FE logistic model. For a non-linear model the marginal effect is not the same as the β model coefficient and is dependent upon the covariate of interest (*X*) and the values of (all) other model covariates [50], [51]. The marginal effects so calculated were understood as being (i) statistics calculated from predictions of a previously fit model (in this case, logistic) at fixed values of some covariates and averaging or otherwise integrating over the remaining covariates [21], [28] (ii) the average of discrete or partial changes over all observations [52]; that is, the average of predictions (AME; the default specification in Stata) rather than the predictions at the average of covariates [26], although the latter may also be calculated (as marginal effects at the mean, MEM). Thus, the AME is given by 
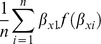
 where 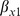 is the estimated log(OR) for variable 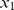, 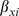 is the logit for the *i*-th observation and 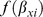 is the probability density function (PDF) of the logistic distribution with regard to 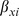 [21]. As noted by Vittinghoff et al [53], the Stata “margins” command estimates potential outcomes (≡ “causal effects”) and provides valid confidence intervals for the parameters of a (in this case, logistic) marginal structural model [54] by averaging over the expected outcome values of the actual and potential values of, say, a binary treatment variable, holding all other covariates fixed at observed values (under the assumption of no residual confounding). We shall refer to these margins of responses (or predictions) as predictive margins after Graubard & Korn [55]. In particular, for a binary covariate *x*, coded (0/1), the marginal mean for *x* = 1 is obtained by considering all the observations of *x* wherever “*x*” appears in the model (for both direct and indirect effects; and similarly for *x* = 0); that is [55]: 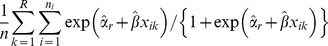 for 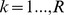, 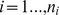, 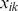 being the covariate effect for the *i*th individual in the *k*th group and 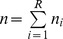 [56], [57]. A point of note with respect to the models estimated in the current paper; predictive margins require that the prediction is a function only of **β**, the 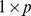 model coefficient vector (matrix) and the independent (fixed) variables, not of stochastic functions (the random effects, 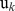).

The following effect computations with 95% CI were undertaken: (i) OR contrasts; using linear predictions via the “predict(xb)” option of the “margins” command and (ii) RR; as the ratio of the provider predictive margins divided by the grand weighted mean of the predictive margins, via nonlinear combination of estimates (the Stata “nlcom” command [58]) and (iii) probability contrasts; the grand weighted mean of the predictive margins was subtracted from the predictive margin for each (ICU) provider. Adjustment of the comparison-wise error rate (individual ICU relative to the grand observation-weighted mean) was based upon the upper limit of the Bonferroni inequality, 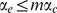, where 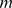 is the comparison number; the adjusted error rate being 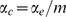 [28], [59], where 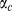 is the comparison-wise error rate and 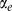 is the experiment-wise error rate.

## Results

The data set consisted of 145355 patient records in 128 ICUs, calendar years 2009 (47.5%) & 2010 (52.5%), with mean(SD) age 60.9(18.8) years, APACHE III score 51.4(28.0) and ICU and hospital mortalities of 7.0% and 10.9% respectively. Fifty six percent were male and 38% were ventilated in the first 24 hours. The mean annual patient volume was 758(404); median 623, range 168–1701. The largest percentage of patients (42.8%) were in tertiary hospitals and resided in the Australian state of New South Wales (31.4%). Patient demographics over calendar year by hospital level and patient surgical status is seen in Table 1. More patients were admitted in 2010 (n = 76346) versus 2009 (n = 69009), but the demographics over the 2 year period were relatively stable.

**Table 1:** Patient demographics by calendar year (2009, 2010).

		Patient surgical status: 2009			Patient surgical status: 2010		
Hospital level		Non-surgical	Elective surgical	Emergency surgical	Non-surgical	Elective surgical	Emergency surgical
Rural	Frequency	6776	1744	1690	7647	1839	1896
	Age (years)	57.9(20.0)	67.1(15.1)	63.4(19.2)	59.0(19.8)	66.9(14.9)	63.9(19.2)
	APIII score	50.2(29.9)	38.2(17.3)	50.6(28.1)	50.1(29.9)	38.1(18.3)	49.7(26.6)
	Male (fraction)	0.56	0.55	0.53	0.56	0.54	0.53
	Ventilation (first 24 hours)	0.25	0.10	0.32	0.23	0.08	0.31
	ICU mortality	0.09	0.00	0.04	0.09	0.01	0.04
	Hospital mortality	0.13	0.02	0.07	0.13	0.02	0.08
Metropolitan	Frequency	8255	2455	2150	8785	2539	2376
	Age (years)	58.1(19.9)	65.5(16.1)	63.8(19.7)	58.5(19.9)	65.5(15.9)	64.1(19.2)
	APIII score	58.6(31.5)	40.7(18.6)	54.7(27.7)	58.8(31.3)	41.2(18.7)	53.1(27.1)
	Male (fraction)	0.53	0.59	0.53	0.54	0.54	0.50
	Ventilation (first 24 hours)	0.38	0.15	0.42	0.36	0.17	0.40
	ICU mortality	0.12	0.01	0.07	0.11	0.01	0.05
	Hospital mortality	0.17	0.04	0.11	0.17	0.03	0.09
Tertiary	Frequency	15699	8378	5369	17704	9047	6002
	Age (years)	55.5(19.7)	63.3(16.0)	58.6(19.8)	55.8(19.7)	62.9(16.2)	58.6(19.9)
	APIII score	64.0(33.0)	43.9(18.0)	56.2(27.7)	61.9(32.2)	43.7(17.6)	54.3(27.1)
	Ventilation (first 24 hours)	0.56	0.50	0.65	0.53	0.50	0.65
	Male (fraction)	0.59	0.61	0.60	0.59	0.60	0.59
	ICU mortality	0.14	0.01	0.08	0.14	0.01	0.07
	Hospital mortality	0.21	0.03	0.14	0.19	0.03	0.13
Private	Frequency	3147	12125	1221	3331	13718	1462
	Age (years)	67.7(17.4)	65.2(15.8)	66.2(17.5)	68.5(16.7)	65.5(16.0)	66.4(18.1)
	APIII score	54.3(27.4)	39.8(18.2)	46.8(24.9)	53.0(26.8)	38.9(16.7)	45.1(22.2)
	Ventilation (first 24 hours)	0.19	0.23	0.27	0.17	0.20	0.26
	Male (fraction)	0.52	0.55	0.47	0.52	0.54	0.45
	ICU mortality	0.09	0.00	0.03	0.09	0.00	0.02
	Hospital mortality	0.15	0.01	0.06	0.15	0.01	0.05

APIII score, APACHE III score. Age and APACHE III score are shown as mean(SD).

Ventilation (first 24 hours), male versus female, ICU and hospital mortality are given as fractions.

**Figure 1:**
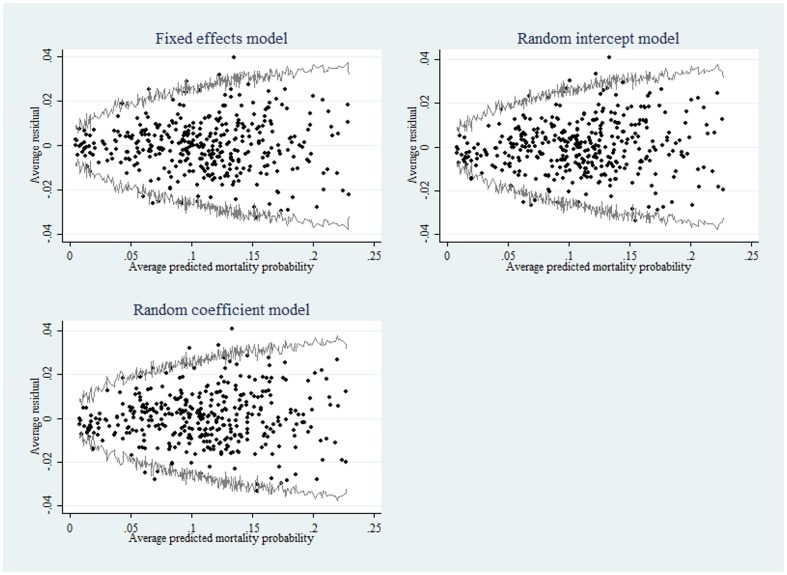
Binned residual plots. Binned residual plots [46] for FE, random intercept and random coefficient models: y-axis, average residual (expectation  = 0); x-axis, average predicted mortality probability.

The fixed effects model (190 parameters, including 127 separate ICU site parameter estimates) had a BIC  = 64057.9, an AUC  = 0.90 and an H-L statistic  = 18.3 (*P* = 0.22; grouping number  = 40). The continuous variable “annual-volume” and the categorical variables “ICU-level” and geographical-location” were identified as collinear and removed from the dependent variable list. When “annual-volume” was parameterised as a decile categorical variable, there was a model AIC increment of 5 (all parameter *P*-values (n = 9) were >0.1) and the variable was not further considered. A global Wald test of the 6 trigonometric seasonality parameters was significant at *P* = 0.004. A random intercept model with the identical independent variable list (excluding the ICU site variable as a categorical variable) had a BIC  = 64457, an AUC  = 0.90 and an H-L statistic  = 41.6 (*p* = 0.32; grouping number  = 40). A random coefficient model (random intercept as ICU site, random coefficient (slope) as centred APACHE III score; unstructured covariance), including the variables “annual-volume” (continuous) and “ICU-level” and “geographical-location” (categorical) had a BIC  = 64555.8, an AUC  = 0.90 and an H-L statistic  = 42.8 (*p* = 0.28; grouping number  = 40). Both RE models satisfied the assumption of normality of random effects estimates (see File S1). Graphical display of the binned residual plots of the three models is seen in Figure 1; in terms of residual percentage outside boundary lines, there was slight advantage for the FE model (3.33%) versus the random intercept (3.84%) and random coefficient (3.85%) models. Overall there was some statistical advantage of the fixed effects model, none the least in terms of computational speed: FE model, 9 seconds; random intercept model, 1.8 hours; random coefficient model, 11.8 hours (computed on a 64-bit PC using an 8-core Intel i7-3960X CPU, clock speed 3.30 GHz). Details of parameter estimates for all three models (fixed effects, random intercept and random coefficient) have been included in File S1.

**Figure 2:**
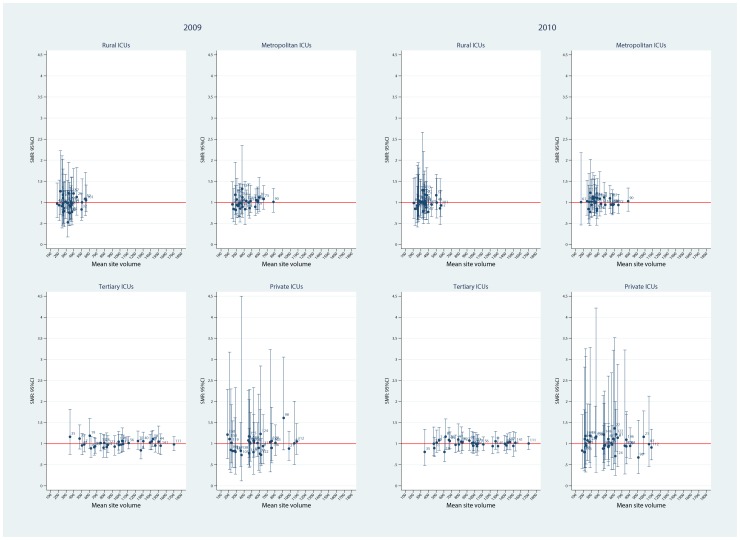
SMR and 95%CI by hospital level and calendar year for fixed effects model. Plots of point SMR (standardised mortality ratio) with 95%CI versus mean (ICU) site volume, by hospital level (rural, metropolitan, tertiary and private) over calendar year (2009, 2010) for the FE model. Null line  = 1.

**Figure 3:**
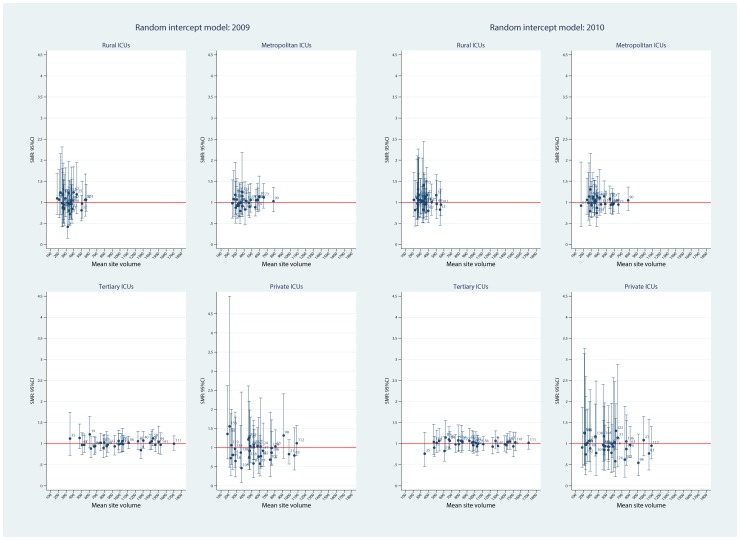
SMR and 95%CI by hospital level and calendar year for random intercept model. Plots of point SMR (standardised mortality ratio) with 95%CI versus mean (ICU) site volume, by hospital level (rural, metropolitan, tertiary and private) over calendar year (2009, 2010) for the random intercept model. Null line  = 1.

**Figure 4:**
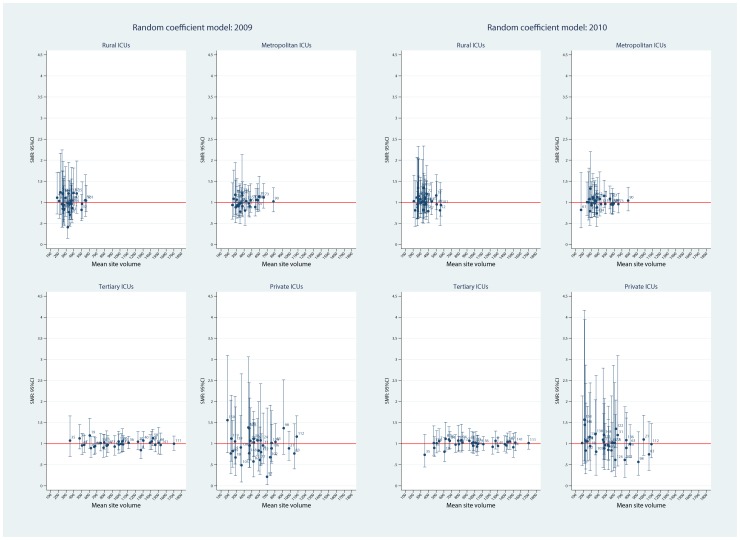
SMR and 95%CI by hospital level and calendar year for random coefficient model. Plots of point SMR (standardised mortality ratio) with 95%CI versus mean (ICU) site volume, by hospital level (rural, metropolitan, tertiary and private) over calendar year (2009, 2010) for the random coefficient model. Null line  = 1.

**Figure 5:**
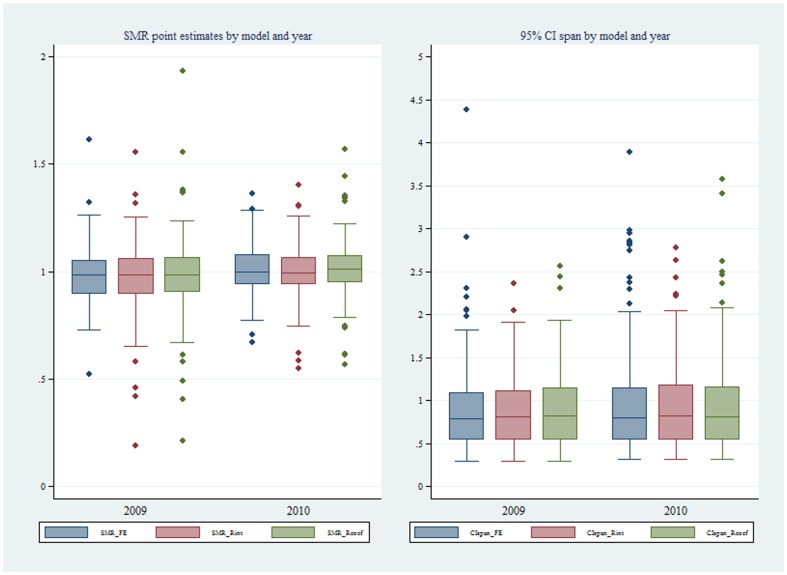
Boxplots of SMR and 95%CI span for different models. Boxplots of: Upper panel, point estimate of SMR (standardised mortality ratio) by model (SMR_FE, SMR for fixed effects model; SMR_Rint, SMR for random intercept model; SMR_Rcoef, SMR for random coefficient model) over year (2009, 2010). Lower panel, 95%CI span by model (CIspan_FE, 95%CI span for fixed effects model; CIspan_Rint, 95%CI span for random intercept model; CIspan_Rcoef, 95%CI span for random coefficient model) over year (2009, 2010).

**Figure 6:**
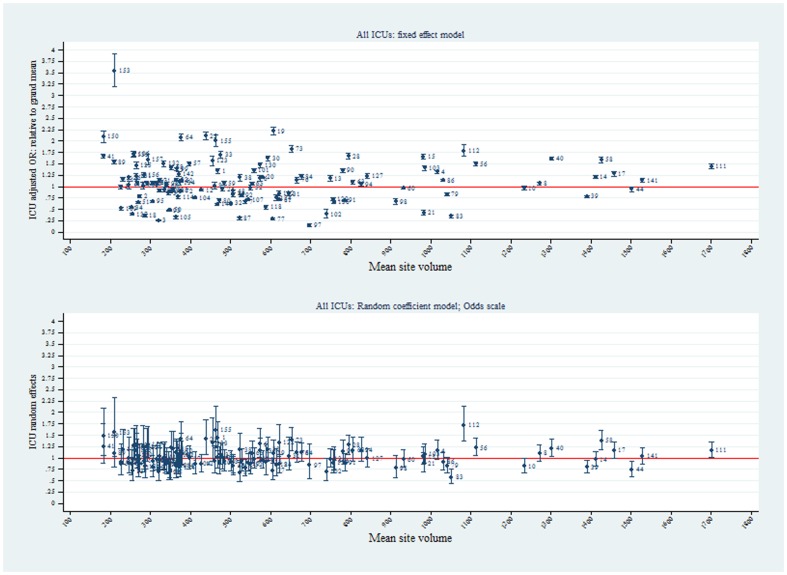
ICU site intercepts (95%CI) on OR and odds scale. FE ICU-site intercepts (95%CI) on OR scale (upper panel) and the ICU-site random effects (95%CI) on odds scale (lower panel) from the random coefficient model. Horizontal axis, mean site volume.

Using the FE model, plots of ICU SMRs and CI by hospital level for the two calendar years, 2009 and 2010, are seen in Figure 2. There was evidence for contraction of the CI spread across the years, more so in the private ICUs. Of interest, no ICU was identified as an outlier with respect to the null ( = 1) in either year. Similarly, for the random intercept and coefficient models, no statistical outliers were identified (Figures 3 and 4, respectively). Box plots of SMR point estimates (left panel) and 95% CI span (right panel) by model and year are seen in Figure 5. Shrinkage of point estimates for all three models is seen, 2010 versus 2009, but no striking difference between models; the random coefficient model having the greater spread of point estimates. Confidence interval span width tended to increase, 2010 versus 2009, and all models displayed “extreme” span widths. A comparison of the (model-based) FE ICU-site intercepts and the ICU-site random effects from the random coefficient model is seen in Figure 6, demonstrating point-estimate shrinkage for the RE model.

**Figure 7:**
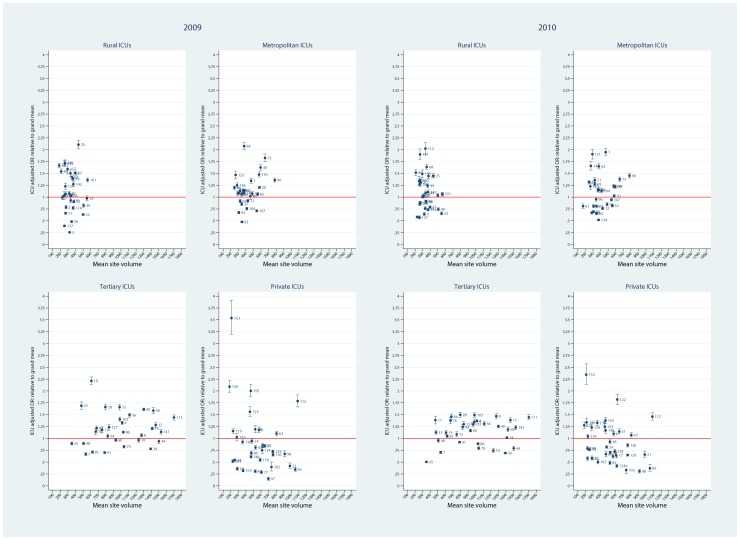
Fixed effects ICU mortality OR (95%CI) by hospital level and calendar year. Plots of predictive ICU mortality OR (95%CI) versus mean (ICU) site volume, by hospital level (rural, metropolitan, tertiary and private) over calendar year (2009, 2010) for the FE model. Grand mean null line  = 1.

**Figure 8:**
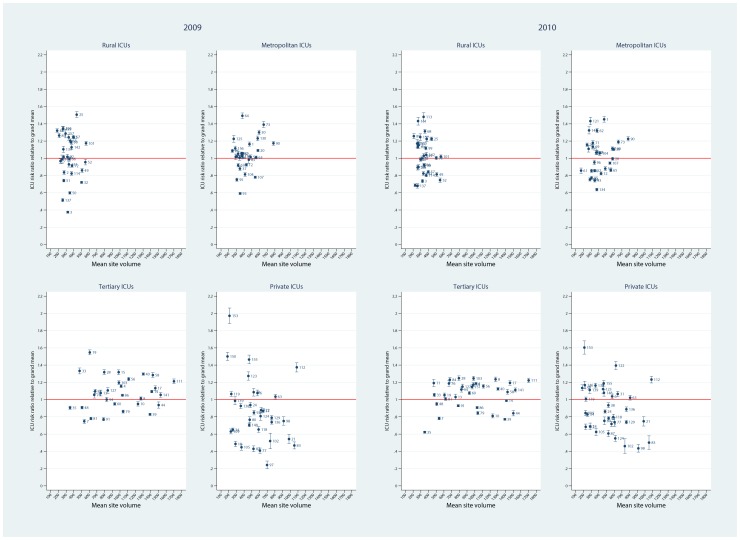
Fixed effects ICU mortality RR (95%CI) by hospital level and calendar year. Plots of predictive ICU mortality risk ratio (RR, 95%CI) versus mean (ICU) site volume, by hospital level (rural, metropolitan, tertiary and private) over calendar year (2009, 2010) for the FE model. Grand mean null line  = 1.

**Figure 9:**
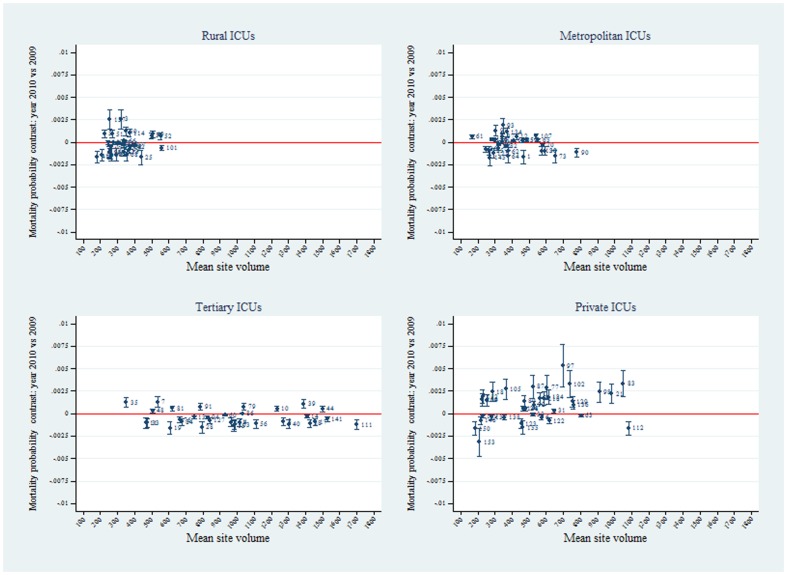
ICU mortality probability contrasts by hospital level and calendar year. Plots of predictive ICU mortality probability contrasts (calendar year 2010 versus 2009) by hospital level (rural, metropolitan, tertiary and private). Bonferroni control of multiple comparisons (see “Statistical analysis”, above).

Predictive margins analysis (OR scale with Bonferroni control of multiple comparisons) of ICUs relative to the grand mean, by hospital level for the calendar years 2009 and 2010 is seen in Figure 7. The spread of the ICU OR estimates relative to the grand mean (y-line  = 1) was seen to decrease over years 2009 to 2010, more evident for tertiary and private ICUs. As an illustration of the versatility of the margins command, we include two further graphics. Figure 8 shows risk ratio estimates relative to the grand mean, by hospital level for the calendar years 2009 and 2010, displaying similar characteristics with respect to the over-year spread of estimates as in Figure 7; and Figure 9, which demonstrates on the probability scale, by hospital level, formal over-year contrasts (calendar year 2010 versus 2009) of the predictive margins with respect to the grand mean, with Bonferroni control of multiple comparisons. A majority of ICUs in the rural / regional, metropolitan and tertiary levels demonstrated a decrease in predicted probability over the 2 calendar years, but in the private sector, this trend was reversed.

## Discussion

Using a fixed-effects logistic model to generate provider mortality probabilities in a large data-base over a two year period we were unable to demonstrate (i) substantive advantage for a conventional random effects approach and (ii) outlier status for any of the ICUs. These findings deserve further comment.

Multiple studies have compared fixed and random effects estimators in assessing provider performance, the key performance indicator usually being the (log)-SMR [5], [17], [18], [60]–[62], although standardisation [63] has not been undertaken in some studies and the user-specific (log)-OR has been advocated [64] and utilised in provider comparison [10], [20], [65]. The calculation of the SMR in the current context is equivalent to indirect standardisation, direct standardisation being “practically impossible when multiple predictors are included in the case-mix adjustment model” [66], and the former method may not sufficiently adjust for case-mix difference / confounding [15], [67]. This being said, formal model development, in terms of appropriate covariates [12]and model assessment has been quite variable in the literature: in particular, the lack of adjustment for mechanical ventilation status, patient transfer, diagnostic categories and seasonality; and little extension beyond reporting of conventional AUC and H-L statistic [68] in terms of model performance. Under the FE model and in the presence of large numbers of providers, for instance >4000 as reported by Ash and co-workers [15], there may be concerns regarding estimator consistency, but such concerns would appear to be more apparent than real [69], [70]. Unlike other studies using a FE approach, we accounted for within-ICU patient correlation using the cluster-variance option of Stata to obtain unbiased variance estimators ([71], see Statistical analysis, above).

A number of papers have suggested that “non-hierarchical” estimators increase the possibility of false outlier classification [10], [11], [67], and the shrinkage of RE estimators has been accepted as a virtue in that it would result in a “…more accurate estimate of a provider's unobserved true performance…” [11], although there has been disquiet at the very consequences of this feature [13], [14]. In the current study, as shown in Figure 3, we were unable to demonstrate this reported characteristic of RE models compared with FE, although shrinkage of the point estimates of the RE intercepts compared with the FE ICU-site intercepts was quite evident (Figure 6), albeit with wide 95%CI (on the odds scale). Apropos this point of contention, a recent combined simulation and empirical study has reported the FE estimator to provide “…high power to identify providers with exceptional outcomes or to estimate the magnitude of the difference from expected for such exceptional providers…” [16]. One aspect of the current study that may inform the lack of demonstration of mortality outliers on the SMR scale was the database that we utilized; a binational Intensive Care data base which was different from that of, say, the COPSS-CMS authors [15] and other papers where specific medical diagnoses in a variety of general hospitals with quite variable (and small) cluster size were addressed. Minimum annualised ICU volume was modest at 168 patients (equivalent to 3 ICU admissions per week); that is, there were no extreme outliers with respect to ICU “cluster” size, although the number of clusters was adequate [72]. We have previously drawn attention to the implications of these particular (Australia and New-Zealand) intra-hospital ICU characteristics when addressing the volume-outcome question [30]. The recent findings of Madigan et al, that “clinical studies that use observational databases can be sensitive to the choice of database” gives credence to such cautions [73].

**Figure 10:**
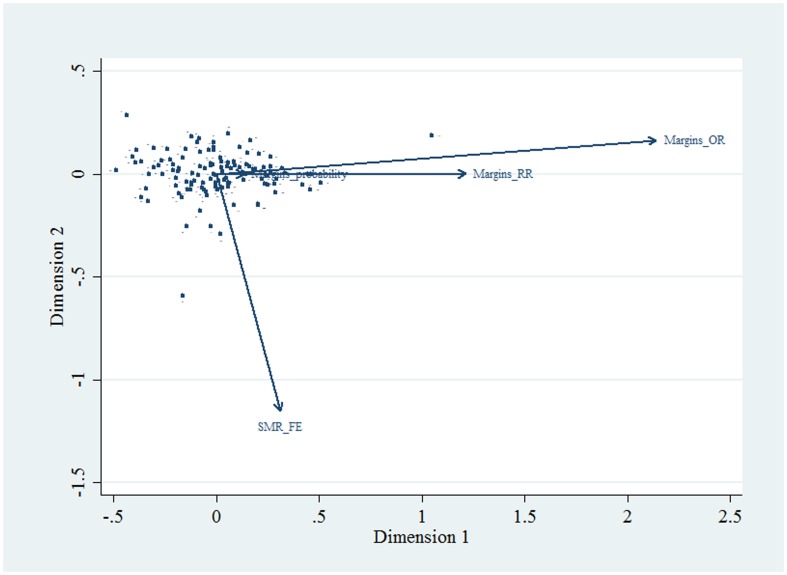
Biplot of multivariate estimate relationships. Biplot demonstrating multivariate relationships between variables (fixed effects SMR (SMR_FE), predictive margins probability (Margins_probability), predictive margins odds ratio (Marginns_OR0 and predictive margins risk ratio (Margins_RR)) represented by arrow-headed lines; observations are represented by “dots” (see “Statistical analysis”, above).

The current study would appear to be one of the first to assess provider performance exploiting predictive margins, the use of the which has distinct advantages: the ability to resolve problems inherent in prediction across categorical predictors (for example, the prediction of the “average” gender effect [74]), that is the average effect versus the effect at the average covariate value, or AME versus MEM (see the discussion of the AME in Statistical analysis, above); seamless computation of ICU-effects with respect to the grand mean on both the OR and RR scale; the ability to formally estimate year-to-year changes in (predicted) mortality as precisely displayed in Figure 9 where over-year changes are seen in tertiary (decrease) and private (increase) ICUs; the ability to define general risk levels in ICU strata, this being evident in the general increase in RR for tertiary ICUs (Figure 8), a finding consistent with our previous observations for ventilated patients in this database [30]; and the computationally simple use of adjustment for multiple comparisons within a modelling framework. An extension of the latter (not presented in the current study) would be the vexed problem of specific between-provider comparisons in so-called caterpillar plots [75]–[77]; within the margins framework this may be easily accomplished using pairwise comparisons (the Stata “pwcompare” module, with say, Bonferroni adjustment [78]). Inference from these model based estimates is thus of some value in assessing provider performance and may be contrasted with, and are orthogonal to (see Figure 10), that provided by the SMR, the statistical properties of which (for example, variance estimation), being non-model based, are somewhat problematic [12], [79]. The SMR is a dimensionless measure of provider outcome and is therefore valid for direct comparisons across providers. Indeed, the SMR may be regarded as the ‘canonical residual provider effect’ and should be uncorrelated with the model-based estimates.

The statistical advantages of the FE approach compared with the RE were quite modest, although computational speed and simplicity recommended the former. As noted in Results, above, ICU level (and geographical region) were unable to be explicitly fitted in the FE model due to confounding / collinearity, but were “recovered” within the model based predictive margins analysis. Such confounding does not arise with RE modelling and FE and RE modelling approaches might be best characterised as complementary, rather than comparative.

Conventional one-stage RE (and FE) estimation considers both “usually” and “unusually” performing providers, leading to inflated random effect variance estimates and the inability to properly account for the latter provider-type (“unusual”) in estimation. A staged approach to estimation which includes a "null" model describing the behaviour of "usual" providers would appear to be apposite [12]; such an approach may also accommodate over-time analysis [80]. Similarly, the simplistic claim that the shrinkage process of RE estimators mitigate against multiple comparisons [67], elides the real problems of false discovery rate and regression to the mean, both of which must be formally handled within a RE scenario [12]. As with the FE estimator, specific requirements of the RE estimator are rarely tested; the distribution of the RE, as reflected in the gradient function [81], and (for the intercept only RE model) lack of correlation between random intercepts and patient case-mix [15], [82]. In the presence of such a correlation, which it is plausible to think may commonly occur, the performance of the RE estimator is “…adversely affected…” [16].

The developed FE model had advantage compared with the conventional RE models and disclosed no ICU performance outliers in calendar years 2009–2010. Current developments in RE estimation, which embrace a “null” model and adjust for the false discovery rate and regression to the mean, are superior to a single application of a RE (or FE) model, but are considerably more complex statistically and computationally. Analysis using predictive margins allows substantial inferential insight into provider performance.

## Supporting Information

File S1 **This file contains supporting information including Table S1-Table S3, Figure S1, and Figure S2.** Table S1, Model estimates: fixed effects. Table S2, Model estimates: random intercept. Table S3, Model estimates: random coefficient. Figure S1, Standardized normal probability plots (P–P plot) of the random effects; random intercept model. Figure S2, Standardized normal probability plots (P–P plot) of the random effects; random coefficient model. a) Random effects for ICU site: APACHE III score. b) Random effects for ICU site: constant.
(DOCX)

## References

[1] Normand S-LT, Shahian DM (2007) Statistical and Clinical Aspects of Hospital Outcomes Profiling. Statistical Science 22: 206–226.

[2] Shahian DM, Normand SL (2008) Comparison of “risk-adjusted” hospital outcomes. Circulation 117: 1955–1963. 18391106 10.1161/CIRCULATIONAHA.107.747873

[3] van Gestel YRBM, Lemmens VEPP, Lingsma HF, de Hingh IHJT, Rutten HJT, et al (2012) The Hospital Standardized Mortality Ratio Fallacy A Narrative Review. Med Care 50: 662–667. 22410410 10.1097/MLR.0b013e31824ebd9f

[4] Iezzoni LI (1997) The risks of risk adjustment. JAMA 278: 1600–1607. 9370507 10.1001/jama.278.19.1600

[5] Kipnis P, Escobar GJ, Draper D (2010) Effect of Choice of Estimation Method on Inter-Hospital Mortality Rate Comparisons. Med Care 48: 458–465. 20393365 10.1097/MLR.0b013e3181d5fe8f

[6] DeLong ER, Peterson ED, DeLong DM, Muhlbaier LH, Hackett S, et al (1997) Comparing risk-adjustment methods for provider profiling. Stat Med 16: 2645–2664. 9421867 10.1002/(sici)1097-0258(19971215)16:23<2645::aid-sim696>3.0.co;2-d

[7] Normand S-LT, Glickman ME, Gatsonis CA (1997) Statistical methods for profiling providers of medical care: issues and applications. Journal of the American Statistical Association 92: 803–814.

[8] Concato J, Feinstein AR, Holford TR (1993) The Risk of Determining Risk with Multivariable Models. Ann Intern Med 118: 201–210. 8417638 10.7326/0003-4819-118-3-199302010-00009

[9] Christiansen CL, Morris CN (1997) Improving the statistical approach to health care provider profiling. Ann Intern Med 127: 764–768. 9382395 10.7326/0003-4819-127-8_part_2-199710151-00065

[10] Moore L, Hanley JA, Turgeon AF, Lavoie A (2010) Evaluating the Performance of Trauma Centers: Hierarchical Modeling Should be Used. Journal of Trauma-Injury Infection and Critical Care 69: 1132–1137. 10.1097/TA.0b013e3181cc8449 20404760

[11] Shahian DM, Torchiana DF, Shemin RJ, Rawn JD, Normand SL (2005) Massachusetts Cardiac Surgery Report Card: Implications of Statistical Methodology. The Annals of Thoracic Surgery 80: 2106–2113. 16305853 10.1016/j.athoracsur.2005.06.078

[12] Kasza J, Moran JL, Solomon PJ (2013) Evaluating the performance of Australian and New Zealand intensive care units in 2009 and 2010. Stat Med 13: 3720–3736. 10.1002/sim.5779 23526209

[13] Mukamel DB, Glance LG, Dick AW, Osler TM (2010) Measuring Quality for Public Reporting of Health Provider Quality: Making It Meaningful to Patients. Am J Public Health 100: 264–269. 20019317 10.2105/AJPH.2008.153759 PMC2804637

[14] Silber JH, Rosenbaum PR, Brachet TJ, Ross RN, Bressler LJ, et al (2010) The Hospital Compare Mortality Model and the Volume-Outcome Relationship. Health Serv Res 45: 1148–1167. 20579125 10.1111/j.1475-6773.2010.01130.x PMC2965498

[15] Ash AS, Fienberg SE, Louis TA, Norman SL, Stukel TA, et al.. (2012) Statistical issues in assessing hospital performance. Committee of Presidents of Statistical Societies.

[16] Kalbfleisch J, Wolfe R (2013) On Monitoring Outcomes of Medical Providers. Stat Biosci 1–17.

[17] Cohen ME, Dimick JB, Bilimoria KY, Ko CY, Richards K, et al (2009) Risk Adjustment in the American College of Surgeons National Surgical Quality Improvement Program: A Comparison of Logistic Versus Hierarchical Modeling. J Am Coll Surg 209: 687–693. 19959035 10.1016/j.jamcollsurg.2009.08.020

[18] Alexandrescu R, Jen MH, Bottle A, Jarman B, Aylin P (2011) Logistic Versus Hierarchical Modeling: An Analysis of a Statewide Inpatient Sample. J Am Coll Surg 213: 392–401. 21784667 10.1016/j.jamcollsurg.2011.06.423

[19] Hannan ELP, Wu CM, DeLong ERP, Raudenbush SWE (2005) Predicting Risk-Adjusted Mortality for CABG Surgery: Logistic Versus Hierarchical Logistic Models. Med Care 43: 726–735. 15970789 10.1097/01.mlr.0000167802.27044.44

[20] Austin PC, Alter DA, Tu JV (2003) The use of fixed- and random-effects models for classifying hospitals as mortality outliers: a Monte Carlo assessment. Med Decis Making 23: 526–529. 14672113 10.1177/0272989X03258443

[21] Mood C (2010) Logistic Regression: Why We Cannot Do What We Think We Can Do, and What We Can Do About It. Eur Sociol Rev 26: 67–82.

[22] Angrist JD (2001) Estimation of limited dependent variable models with dummy endogenous regressors: Simple strategies for empirical practice. Journal of Business & Economic Statistics 19: 2–16.

[23] Stow PJ, Hart GK, Higlett T, George C, Herkes R, et al (2006) Development and implementation of a high-quality clinical database: the Australian and New Zealand Intensive Care Society Adult Patient Database. J Crit Care 21: 133–141. 16769456 10.1016/j.jcrc.2005.11.010

[24] Australian and New Zealand Intensive Care Society (2013) Centre for Outcome and Resource Evaluation (ANZICS CORE). Available: http://www.anzics.com.au/core. Accessed 2013 Jan 20.

[25] Cameron CC, Trivedi PK (2010) Nonlinear regression methods. In: Microeconomics using Stata. College Station, Texas: Stata Press. pp. 319–362.

[26] Williams R (2012) Using the margins command to estimate and interpret adjusted predictions and marginal effects. Stata Journal 12: 308–331.

[27] Muller CJ, MacLehose RF (2014) Estimating predicted probabilities from logistic regression: different methods correspond to different target populations. Int J Epidemiol. 10.1093/ije/dyu029 PMC4052139 24603316

[28] Stata Corporation (2013) Margins - Marginal means, predictive margins, and marginal effects. Available: http://www.stata.com/manuals13/margins.pdf. Accessed 2013 Sep 14.

[29] Moran JL, Bristow P, Solomon PJ, George C, Hart GK, et al (2008) Mortality and length-of-stay outcomes, 1993-2003, in the binational Australian and New Zealand intensive care adult patient database. Crit Care Med 36: 46–61. 18090383 10.1097/01.CCM.0000295313.08084.58

[30] Moran JL, Solomon PJ (2012) Mortality and Intensive Care volume in ventilated patients, 1995-2009, in the Australian and New Zealand bi-national adult patient intensive care database. Crit Care Med 40: 800–812. 22080640 10.1097/CCM.0b013e318236f2af

[31] Knaus WA, Wagner DP, Draper EA, Zimmerman JE, Bergner M, et al (1991) The APACHE III prognostic system. Risk prediction of hospital mortality for critically ill hospitalized adults. Chest 100: 1619–1636. 1959406 10.1378/chest.100.6.1619

[32] Wagner D, Knaus W, Bergner M (1989) Statistical-Methods. Crit Care Med 17: S194–S198. 2591241

[33] ANZICS Centre for Outcome and Resource Evaluation (CORE) of the Australian and New Zealand Intensive Care Society (ANZICS) (2012) APD Data Dictionary: Version 3.2 Updated February 2012. Available: http://www.anzics.com.au/core/data-collection-tools. Accessed 2012 Sep 21.

[34] Kahn JM, Goss CH, Heagerty PJ, Kramer AA, O'Brien CR, et al (2006) Hospital Volume and the Outcomes of Mechanical Ventilation. N Engl J Med 355: 41–50. 16822995 10.1056/NEJMsa053993

[35] Gunasekara FI, Richardson K, Carter K, Blakely T (2014) Fixed effects analysis of repeated measures data. Int J Epidemiol 43: 264–269. 24366487 10.1093/ije/dyt221

[36] Rogers W (1993) sg17: Regression standard errors in clustered samples. Stata Technical Bulletin Reprints 3: 88–94.

[37] Stata Corporation (2013) Estimation and postestimation commands. Available: http://www.stata.com/manuals13/u20.pdf. Accessed 2013 Sep 14.

[38] Angrist JD, Pischke J-S (2009) Nonstandard error issues. In: Mostly Harmless Econometrics: An Empiricist's Companion. Princeton, NJ: Princeton University Press. pp. 293–323.

[39] Rabe-Hesketh S, Skrondal A, Pickles A (2002) Reliable estimation of generalized linear mixed models using adaptive quadrature. Stata Journal 2: 1–21.

[40] Stolwijk AM, Straatman H, Zielhuis GA (1999) Studying seasonality by using sine and cosine functions in regression analysis. J Epidemiol Community Health 53: 235–238. 10396550 10.1136/jech.53.4.235 PMC1756865

[41] Harrell FE Jr. (2001) Regression modelling strategies: with applications to linear models, logistic regression, and survival analysis. New York: Springer-Verlag.

[42] Stata Corporation (2013) _rmcoll — Remove collinear variables. Available: http://www.stata.com/manuals13/p.pdf. Accessed 2013 Sep 14.

[43] Rowan KM, Kerr JH, Major E, McPherson K, Short A, et al (1993) Intensive Care Society's APACHE II study in Britain and Ireland—I: Variations in case mix of adult admissions to general intensive care units and impact on outcome. BMJ 307: 972–977. 8241908 10.1136/bmj.307.6910.972 PMC1679155

[44] Paul P, Pennell ML, Lemeshow S (2013) Standardizing the power of the Hosmer−Lemeshow goodness of fit test in large data sets. Stat Med 32: 67–80. 22833304 10.1002/sim.5525

[45] Wilk MB, Gnanades R (1968) Probability Plotting Methods for Analysis of Data. Biometrika 55: 1–17. 5661047

[46] Gelman A, Hill J (2007) Logistic Regression. In: Data analysis using Regression and Multilelvel/ Hierarchal Models. New York, NY: Cambridge University Press. pp. 79–108.

[47] O'Brien BJ, Drummond MF, Labelle RJ, Willan A (1994) In search of power and significance: issues in the design and analysis of stochastic cost-effectiveness studies in health care. Med Care 32: 150–163. 8302107 10.1097/00005650-199402000-00006

[48] Gabriel KR, Odoroff CL (1990) Biplots in medical research. Stat Med 9: 469–485. 2349401 10.1002/sim.4780090502

[49] Moran J, Solomon P (2010) Australian and New Zealand Intensive Care Society (ANZICS) (2010) Global quantitative indices reflecting provider process-of-care: data-base derivation. BMC Medical Research Methodology 10: 32. 20398426 10.1186/1471-2288-10-32 PMC2873511

[50] Mustillo S, Landerman LR, Land KC (2012) Modeling Longitudinal Count Data: Testing for Group Differences in Growth Trajectories Using Average Marginal Effects. Sociological Methods & Research 41: 467–487.

[51] Norton EC (2012) Log odds and ends. NBER Working Paper Series. Available: http://www.nber.org/papers/w18252. Accessed 2013 Oct 2.

[52] Bartus T (2005) Estimation of marginal effects using margeff. Stata Journal 5: 309–329.

[53] Vittinghoff E, Glidden DV, Shiboski SC, McCulloch CE (2012) Strengthening causal inference. In: Vittinghoff E, Glidden DV, Shiboski SC, McCulloch CE, editors. Regression methods in Biostatistics: Linear, logistic, suvival and repeated measures models. New York: Springer Science+Business Media, LLC. pp. 331–394.

[54] Clarke PS, Windmeijer F (2010) Identification of causal effects on binary outcomes using structural mean models. Biostat 11: 756–770. 10.1093/biostatistics/kxq024 PMC4161996 20522728

[55] Graubard BI, Korn EL (1999) Predictive margins with survey data. Biometrics 55: 652–659. 11318229 10.1111/j.0006-341x.1999.00652.x

[56] Chang IM, Gelman R, Pagano M (1982) Corrected Group Prognostic Curves and Summary Statistics. J Chronic Dis 35: 669–674. 7096530 10.1016/0021-9681(82)90019-4

[57] Lane PW, Nelder JA (1982) Analysis of Covariance and Standardization as Instances of Prediction. Biometrics 38: 613–621. 7171691

[58] Stata Corporation (2013) nlcom - Nonlinear combinations of estimators. Available: http://www.stata.com/manuals13/r.pdf. Accessed 2013 Sep 14.

[59] Mitchell MN (2012) Categorical predictors. In: Mitchell MN, editors.Interpreting and Visualizing Regression Models Using Stata. College Station, TX: Stata Press. pp. 167–208.

[60] Krumholz HM, Wang Y, Mattera JA, Wang YF, Han LF, et al (2006) An administrative claims model suitable for profiling hospital performance based on 30-day mortality rates among patients with heart failure. Circulation 113: 1693–1701. 16549636 10.1161/CIRCULATIONAHA.105.611194

[61] Glance LG, Dick A, Osler TM, Li Y, Mukamel DB (2006) Impact of changing the statistical methodology on hospital and surgeon ranking: the case of the New York State cardiac surgery report card. Med Care 44: 311–319. 16565631 10.1097/01.mlr.0000204106.64619.2a

[62] Glance LG, Dick AW, Osler TM, Mukamel D (2003) Using hierarchical modeling to measure ICU quality. Intensive Care Med 29: 2223–2229. 14534777 10.1007/s00134-003-1959-9

[63] Chan CK, Feinstein AR, Jekel JF, Wells CK (1988) The Value and Hazards of Standardization in Clinical Epidemiologic Research. J Clin Epidemiol 41: 1125–1134. 3256304 10.1016/0895-4356(88)90082-0

[64] Grunkemeier GL, Wu Y (2007) What are the odds? Ann Thorac Surg 83: 1240–1244. 17383319 10.1016/j.athoracsur.2006.12.080

[65] Sanagou M, Wolfe R, Forbes A, Reid CM (2012) Hospital-level associations with 30-day patient mortality after cardiac surgery: a tutorial on the application and interpretation of marginal and multilevel logistic regression. BMC Medical Research Methodology 12. 10.1186/1471-2288-12-28 PMC3366874 22409732

[66] Pouw ME, Peelen LM, Lingsma HF, Pieter D, Steyerberg E, et al (2013) Hospital standardized mortality ratio: consequences of adjusting hospital mortality with indirect standardization. Plos One 8: e59160. 23593133 10.1371/journal.pone.0059160 PMC3621877

[67] Mohammed MA, Manktelow BN, Hofer TP (2012) Comparison of four methods for deriving hospital standardised mortality ratios from a single hierarchical logistic regression model. Stat Methods Med Res. 10.1177/0962280212465165 23136148

[68] Harrison DAP, Brady ARM, Parry GJP, Carpenter JRD, Rowan KD (2006) Recalibration of risk prediction models in a large multicenter cohort of admissions to adult, general critical care units in the United Kingdom. Crit Care Med 34: 1378–1388. 16557153 10.1097/01.CCM.0000216702.94014.75

[69] Greene WH (2001) Estimating Econometric Models With Fixed Effects. Available: http://www.stern.nyu.edu/eco/wkpapers/workingpapers01/01-10Greene.doc. Accessed 2010 Oct 13.

[70] Mroz TA, Zayats YV (2008) Arbitrarily Normalized Coefficients, Information Sets, and False Reports of Biases in Binary Outcome Models. Rev Econ Stat 90: 406–413.

[71] Williams RL (2000) A Note on Robust Variance Estimation for Cluster-Correlated Data. Biometrics 56: 645–646. 10877330 10.1111/j.0006-341x.2000.00645.x

[72] Bryan ML, Jenkins SP (2013) Regression analysis of country effects using multilevel data: a cautionary tale. ISER Working Paper Series: 2013–14. Available: https://www.iser.essex.ac.uk/publications/working-papers/iser/2013-14; Accessed 2013 Sep 22.

[73] Madigan D, Ryan PB, Schuemie M, Stang PE, Overhage JM, et al.. (2013) Evaluating the Impact of Database Heterogeneity on Observational Study Results. Am J Epidemiol. 10.1093/aje/kwt010 PMC3736754 23648805

[74] MacKenzie TA, Brown JR, Likosky DS, Wu Y, Grunkemeier GL (2012) Review of Case-Mix Corrected Survival Curves. Ann Thorac Surg 93: 1416–1425. 22541174 10.1016/j.athoracsur.2011.12.094

[75] Goldstein H, Healy MJR (1995) The graphical presentation of a collection of means. Journal of the Royal Statistical Society, A 158: 175–177.

[76] Mohammed MA, Deeks JJ (2008) In the Context of Performance Monitoring, the Caterpillar Plot Should Be Mothballed in Favor of the Funnel Plot. The Annals of Thoracic Surgery 86: 348. 18573460 10.1016/j.athoracsur.2007.10.028

[77] Spiegelhalter D (2002) Funnel plots for institutional comparison. Quality & Safety in Health Care 11: 390–391. 10.1136/qhc.11.4.390-a PMC1757996 12468705

[78] Stata Corporation (2013) pwcompare - Pairwise comparisons. Available: http://www.stata.com/manuals13/r.pdf. Accessed 2013 Sep 14.

[79] Hosmer DW, Lemeshow S (1997) Confidence interval estimates of an index of quality performance based on logistic regression models - Reply. Stat Med 16: 1303. 8552894 10.1002/sim.4780141909

[80] Solomon PJ, Kasza J, Moran JL (2014) Identifying unusual performance in Australian and New Zealand intensive care units from 2000 to 2010. BMC Medical Research Methodology 14. 10.1186/1471-2288-14-53 PMC4021168 24755369

[81] Verbeke G, Molenberghs G (2013) The gradient function as an exploratory goodness-of-fit assessment of the random-effects distribution in mixed models. Biostat doi:10.1093/biostatistics/kxs059. 10.1093/biostatistics/kxs059 23376427

[82] Fielding A (2004) The role of the Hausman test and whether higher level effects should be treated as random or fixed. Multilevel Modelling Newsletter 16. Available: http://www.cmm.bristol.ac.uk/learning-training/multilevel-m-support/new16-2.pdf. Accessed 2010 Mar 6.

