# Supplementary material for: Fixed Effects Modelling for Provider Mortality Outcomes: Analysis of the Australia and New Zealand Intensive Care Society (ANZICS) Adult Patient Data-Base
Source: PLoS One. 2014 Jul 16;9(7):e102297. doi: 10.1371/journal.pone.0102297 (PMC4100889; doi:10.1371/journal.pone.0102297)
Supplement: File S1 — This file contains supporting information including Table S1-Table S3, Figure S1, and Figure S2. Table S1, Model estimates: fixed effects. Table S2, Model estimates: random intercept. Table S3, Model estimates: random coefficient. Figure S1, Standardized normal probability plots (P–P plot) of the random effects; random intercept model. Figure S2, Standardized normal probability plots (P–P plot) of the random effects; random coefficient model. a) Random effects for ICU site: APACHE III score. b) Random effects for ICU site: constant. (DOCX) [file pone.0102297.s001.docx]

Table S1. Model estimates: fixed effects

| **Parameter** | **Estimate** | **P-value** | **Lower 95%CI** | **Upper 95%CI** |
| --- | --- | --- | --- | --- |
| Age: years | 1.017 | 0.000 | 1.015 | 1.018 |
| Age squared | 1.000 | 0.001 | 1.000 | 1.000 |
| Ventilation | 1.464 | 0.000 | 1.351 | 1.587 |
| APACHE III score: centered | 1.064 | 0.000 | 1.060 | 1.068 |
| APACHE III score squared | 1.000 | 0.000 | 1.000 | 1.000 |
| Ventilation x APACHE III score | 0.987 | 0.000 | 0.983 | 0.990 |
| Ventilation x APACHE III score squared | 1.000 | 0.002 | 1.000 | 1.000 |
| *Non-surgical diagnostic category*  Cardiovascular_medical | 1.000 |  | 1.000 | 1.000 |
| Respiratory medical | 1.298 | 0.000 | 1.138 | 1.482 |
| Liver_Gastro-intestinal_medical | 0.626 | 0.000 | 0.529 | 0.740 |
| Central nervous system: medical | 1.556 | 0.000 | 1.317 | 1.840 |
| Sepsis | 0.727 | 0.000 | 0.637 | 0.829 |
| Trauma | 0.560 | 0.000 | 0.469 | 0.669 |
| Metabolic Hormonal | 0.149 | 0.000 | 0.118 | 0.188 |
| Haematologic | 1.441 | 0.017 | 1.066 | 1.948 |
| Renal_Genito-urinary system | 0.473 | 0.000 | 0.364 | 0.613 |
| Other medical disorders | 0.610 | 0.007 | 0.426 | 0.874 |
| *Surgical diagnostic category*  Cardio-Vascular elective | 0.149 | 0.000 | 0.121 | 0.184 |
| Thoracic elective | 0.400 | 0.000 | 0.312 | 0.513 |
| Gastro-intestinal elective | 0.382 | 0.000 | 0.320 | 0.457 |
| Central nervous system elective | 0.484 | 0.000 | 0.336 | 0.697 |
| Traumatic/Orthopaedic elective | 0.210 | 0.000 | 0.160 | 0.275 |
| Renal_Genito-urinary system elective | 0.126 | 0.000 | 0.083 | 0.189 |
| Gynaecological/Oncological/Hormonal elective | 0.101 | 0.000 | 0.057 | 0.179 |
| Cardio-Vascular emergency | 0.446 | 0.000 | 0.355 | 0.560 |
| Thoracic emergency | 0.665 | 0.018 | 0.474 | 0.931 |
| Gastro-intestinal emergency | 0.517 | 0.000 | 0.444 | 0.602 |
| Central nervous system emergency | 1.647 | 0.000 | 1.368 | 1.983 |
| Traumatic/Orthopaedic emergency | 0.456 | 0.000 | 0.379 | 0.548 |
| Renal_Genito-urinary system emergency | 0.257 | 0.000 | 0.151 | 0.439 |
| Gynaecological/Oncological/Hormonal emergency | 0.155 | 0.000 | 0.074 | 0.322 |
| Cardiovascular_medical x APACHE III score | 1.000 |  | 1.000 | 1.000 |
| Respiratory medical x APACHE III score | 0.992 | 0.000 | 0.989 | 0.995 |
| Liver_Gastro-intestinal_medical x APACHE III score | 1.006 | 0.005 | 1.002 | 1.010 |
| Central nervous system: medical x APACHE III score | 0.998 | 0.250 | 0.993 | 1.002 |
| Sepsis x APACHE III score | 0.999 | 0.351 | 0.996 | 1.002 |
| Trauma x APACHE III score | 1.016 | 0.000 | 1.011 | 1.021 |
| Metabolic Hormonal x APACHE III score | 1.006 | 0.020 | 1.001 | 1.011 |
| Haematologic x APACHE III score | 0.993 | 0.070 | 0.986 | 1.001 |
| Renal_Genito-urinary system x APACHE III score | 0.994 | 0.059 | 0.988 | 1.000 |
| Other medical disorders x APACHE III score | 1.002 | 0.739 | 0.993 | 1.011 |
| Cardio-Vascular elective x APACHE III score | 1.008 | 0.031 | 1.001 | 1.014 |
| Thoracic elective x APACHE III score | 0.997 | 0.425 | 0.989 | 1.005 |
| Gastro-intestinal elective x APACHE III score | 0.995 | 0.106 | 0.990 | 1.001 |
| Central nervous system elective x APACHE III score | 0.994 | 0.410 | 0.980 | 1.008 |
| Traumatic/Orthopaedic elective x APACHE III score | 1.007 | 0.338 | 0.993 | 1.021 |
| Renal_Genito-urinary system elective x APACHE III score | 1.003 | 0.664 | 0.990 | 1.016 |
| Gynaecological/Oncological/Hormonal elective x APACHE III score | 1.021 | 0.251 | 0.985 | 1.058 |
| Cardio-Vascular emergency x APACHE III score | 1.003 | 0.192 | 0.998 | 1.008 |
| Thoracic emergency x APACHE III score | 0.995 | 0.310 | 0.986 | 1.004 |
| Gastro-intestinal emergency x APACHE III score | 0.998 | 0.171 | 0.994 | 1.001 |
| Central nervous system: emergency x APACHE III score | 0.995 | 0.079 | 0.990 | 1.001 |
| Traumatic/Orthopaedic emergency x APACHE III score | 1.011 | 0.000 | 1.006 | 1.017 |
| Renal_Genito-urinary system emergency x APACHE III score | 0.997 | 0.656 | 0.982 | 1.012 |
| Gynaecological/Oncological/Hormonal emergency x APACHE III score | 1.008 | 0.495 | 0.986 | 1.030 |
| No transfer to ICU | 1.000 |  | 1.000 | 1.000 |
| Inter-hospital transfer | 0.813 | 0.000 | 0.750 | 0.881 |
| Direct ICU-ICU transfer | 0.968 | 0.745 | 0.798 | 1.175 |
| ICU site 1 | 1.000 |  | 1.000 | 1.000 |
| ICU site 2 | 0.632 | 0.000 | 0.603 | 0.663 |
| ICU site 3 | 0.282 | 0.000 | 0.276 | 0.288 |
| ICU site 4 | 0.821 | 0.000 | 0.802 | 0.841 |
| ICU site 5 | 0.462 | 0.000 | 0.445 | 0.480 |
| ICU site 6 | 0.694 | 0.000 | 0.657 | 0.734 |
| ICU site 7 | 0.417 | 0.000 | 0.400 | 0.435 |
| ICU site 8 | 0.798 | 0.000 | 0.768 | 0.829 |
| ICU site 9 | 0.369 | 0.000 | 0.341 | 0.399 |
| ICU site 10 | 0.523 | 0.000 | 0.507 | 0.538 |
| ICU site 11 | 0.835 | 0.000 | 0.793 | 0.878 |
| ICU site 12 | 0.513 | 0.000 | 0.495 | 0.532 |
| ICU site 13 | 0.693 | 0.000 | 0.665 | 0.722 |
| ICU site 14 | 0.676 | 0.000 | 0.660 | 0.693 |
| ICU site 15 | 0.912 | 0.000 | 0.890 | 0.934 |
| ICU site 16 | 0.808 | 0.000 | 0.783 | 0.834 |
| ICU site 17 | 0.293 | 0.000 | 0.273 | 0.315 |
| ICU site 18 | 0.991 | 0.615 | 0.959 | 1.025 |
| ICU site 19 | 0.682 | 0.000 | 0.662 | 0.703 |
| ICU site 20 | 0.315 | 0.000 | 0.282 | 0.352 |
| ICU site 21 | 0.641 | 0.000 | 0.622 | 0.659 |
| ICU site 22 | 0.526 | 0.000 | 0.501 | 0.552 |
| ICU site 23 | 1.011 | 0.541 | 0.975 | 1.049 |
| ICU site 24 | 0.775 | 0.000 | 0.753 | 0.797 |
| ICU site 25 | 0.962 | 0.003 | 0.937 | 0.987 |
| ICU site 26 | 0.818 | 0.000 | 0.794 | 0.842 |
| ICU site 27 | 0.562 | 0.000 | 0.536 | 0.590 |
| ICU site 28 | 0.497 | 0.000 | 0.481 | 0.513 |
| ICU site 29 | 0.837 | 0.000 | 0.816 | 0.857 |
| ICU site 30 | 0.396 | 0.000 | 0.379 | 0.415 |
| ICU site 31 | 0.424 | 0.000 | 0.413 | 0.435 |
| ICU site 32 | 0.571 | 0.000 | 0.552 | 0.590 |
| ICU site 33 | 0.510 | 0.000 | 0.491 | 0.531 |
| ICU site 34 | 0.640 | 0.000 | 0.601 | 0.681 |
| ICU site 35 | 0.449 | 0.000 | 0.434 | 0.464 |
| ICU site 36 | 0.868 | 0.000 | 0.848 | 0.888 |
| ICU site 37 | 0.997 | 0.879 | 0.964 | 1.032 |
| ICU site 38 | 0.529 | 0.000 | 0.505 | 0.554 |
| ICU site 39 | 0.565 | 0.000 | 0.546 | 0.585 |
| ICU site 40 | 0.471 | 0.000 | 0.451 | 0.491 |
| ICU site 41 | 0.431 | 0.000 | 0.418 | 0.445 |
| ICU site 42 | 0.469 | 0.000 | 0.455 | 0.483 |
| ICU site 43 | 0.505 | 0.000 | 0.479 | 0.531 |
| ICU site 44 | 0.860 | 0.000 | 0.842 | 0.879 |
| ICU site 45 | 0.681 | 0.000 | 0.658 | 0.704 |
| ICU site 46 | 0.848 | 0.000 | 0.829 | 0.868 |
| ICU site 47 | 0.572 | 0.000 | 0.551 | 0.594 |
| ICU site 48 | 0.649 | 0.000 | 0.630 | 0.667 |
| ICU site 49 | 0.513 | 0.000 | 0.478 | 0.551 |
| ICU site 50 | 0.828 | 0.000 | 0.807 | 0.850 |
| ICU site 51 | 0.659 | 0.000 | 0.625 | 0.695 |
| ICU site 52 | 0.976 | 0.140 | 0.946 | 1.008 |
| ICU site 53 | 0.571 | 0.000 | 0.553 | 0.589 |
| ICU site 54 | 0.587 | 0.000 | 0.572 | 0.603 |
| ICU site 55 | 0.638 | 0.000 | 0.609 | 0.668 |
| ICU site 56 | 0.936 | 0.000 | 0.903 | 0.970 |
| ICU site 57 | 0.643 | 0.000 | 0.619 | 0.668 |
| ICU site 58 | 0.746 | 0.000 | 0.725 | 0.766 |
| ICU site 59 | 0.716 | 0.000 | 0.694 | 0.739 |
| ICU site 60 | 0.975 | 0.213 | 0.937 | 1.015 |
| ICU site 61 | 0.755 | 0.000 | 0.718 | 0.794 |
| ICU site 62 | 0.248 | 0.000 | 0.227 | 0.272 |
| ICU site 63 | 0.493 | 0.000 | 0.476 | 0.510 |
| ICU site 64 | 0.408 | 0.000 | 0.379 | 0.440 |
| ICU site 65 | 0.521 | 0.000 | 0.508 | 0.534 |
| ICU site 66 | 0.696 | 0.000 | 0.677 | 0.716 |
| ICU site 67 | 0.215 | 0.000 | 0.186 | 0.249 |
| ICU site 68 | 0.812 | 0.000 | 0.791 | 0.834 |
| ICU site 69 | 0.576 | 0.000 | 0.542 | 0.612 |
| ICU site 70 | 0.613 | 0.000 | 0.596 | 0.631 |
| ICU site 71 | 0.242 | 0.000 | 0.224 | 0.263 |
| ICU site 72 | 0.942 | 0.000 | 0.916 | 0.969 |
| ICU site 73 | 0.858 | 0.000 | 0.834 | 0.883 |
| ICU site 74 | 0.491 | 0.000 | 0.477 | 0.505 |
| ICU site 75 | 0.462 | 0.000 | 0.440 | 0.484 |
| ICU site 76 | 0.351 | 0.000 | 0.338 | 0.364 |
| ICU site 77 | 0.697 | 0.000 | 0.680 | 0.713 |
| ICU site 78 | 0.419 | 0.000 | 0.406 | 0.432 |
| ICU site 79 | 0.613 | 0.000 | 0.584 | 0.643 |
| ICU site 80 | 0.091 | 0.000 | 0.074 | 0.113 |
| ICU site 81 | 0.295 | 0.000 | 0.266 | 0.328 |
| ICU site 82 | 0.793 | 0.000 | 0.770 | 0.817 |
| ICU site 83 | 0.745 | 0.000 | 0.721 | 0.771 |
| ICU site 84 | 0.218 | 0.000 | 0.175 | 0.272 |
| ICU site 85 | 0.888 | 0.000 | 0.863 | 0.914 |
| ICU site 86 | 0.583 | 0.000 | 0.560 | 0.607 |
| ICU site 87 | 0.266 | 0.000 | 0.242 | 0.292 |
| ICU site 88 | 0.893 | 0.000 | 0.860 | 0.928 |
| ICU site 89 | 0.506 | 0.000 | 0.489 | 0.523 |
| ICU site 90 | 0.389 | 0.000 | 0.365 | 0.414 |
| ICU site 91 | 0.710 | 0.000 | 0.682 | 0.740 |
| ICU site 92 | 0.882 | 0.000 | 0.860 | 0.905 |
| ICU site 93 | 0.993 | 0.853 | 0.926 | 1.066 |
| ICU site 94 | 0.844 | 0.000 | 0.801 | 0.889 |
| ICU site 95 | 0.459 | 0.000 | 0.441 | 0.477 |
| ICU site 96 | 0.622 | 0.000 | 0.583 | 0.664 |
| ICU site 97 | 0.374 | 0.000 | 0.354 | 0.396 |
| ICU site 98 | 0.670 | 0.000 | 0.638 | 0.703 |
| ICU site 99 | 0.883 | 0.000 | 0.839 | 0.929 |
| ICU site 100 | 0.775 | 0.000 | 0.730 | 0.823 |
| ICU site 101 | 0.859 | 0.000 | 0.803 | 0.918 |
| ICU site 102 | 0.357 | 0.000 | 0.331 | 0.385 |
| ICU site 103 | 0.812 | 0.000 | 0.767 | 0.860 |
| ICU site 104 | 0.771 | 0.000 | 0.748 | 0.795 |
| ICU site 105 | 0.416 | 0.000 | 0.396 | 0.438 |
| ICU site 106 | 0.821 | 0.000 | 0.789 | 0.854 |
| ICU site 107 | 0.925 | 0.005 | 0.877 | 0.976 |
| ICU site 108 | 0.651 | 0.000 | 0.621 | 0.684 |
| ICU site 109 | 0.441 | 0.000 | 0.424 | 0.459 |
| ICU site 110 | 0.774 | 0.000 | 0.744 | 0.805 |
| ICU site 111 | 0.462 | 0.000 | 0.438 | 0.487 |
| ICU site 112 | 0.286 | 0.000 | 0.272 | 0.300 |
| ICU site 113 | 0.694 | 0.000 | 0.658 | 0.732 |
| ICU site 114 | 0.692 | 0.000 | 0.649 | 0.737 |
| ICU site 115 | 0.732 | 0.000 | 0.712 | 0.754 |
| ICU site 116 | 0.716 | 0.000 | 0.685 | 0.748 |
| ICU site 117 | 1.035 | 0.197 | 0.982 | 1.089 |
| ICU site 118 | 0.770 | 0.000 | 0.716 | 0.828 |
| ICU site 119 | 0.856 | 0.000 | 0.792 | 0.925 |
| ICU site 120 | 0.525 | 0.000 | 0.496 | 0.556 |
| ICU site 121 | 0.670 | 0.000 | 0.634 | 0.707 |
| ICU site 122 | 1.010 | 0.740 | 0.955 | 1.067 |
| ICU site 123 | 0.817 | 0.000 | 0.786 | 0.849 |
| ICU site 124 | 0.351 | 0.000 | 0.324 | 0.379 |
| ICU site 125 | 1.621 | 0.000 | 1.482 | 1.773 |
| ICU site 126 | 0.969 | 0.276 | 0.915 | 1.026 |
| ICU site 127 | 0.560 | 0.000 | 0.542 | 0.578 |
| ICU site 128 | 0.934 | 0.027 | 0.878 | 0.992 |
| Year 2010 | 0.949 | 0.041 | 0.902 | 0.998 |
| Cosine monthly | 0.969 | 0.722 | 0.814 | 1.153 |
| Sine monthly | 0.994 | 0.953 | 0.810 | 1.219 |
| Sine 6 monthly | 1.034 | 0.026 | 1.004 | 1.064 |
| Cosine 6 monthly | 1.013 | 0.440 | 0.981 | 1.046 |
| Sine weekly | 1.032 | 0.760 | 0.842 | 1.265 |
| Cosine weekly | 0.996 | 0.961 | 0.837 | 1.185 |
| Constant | 0.120 | 0.000 | 0.107 | 0.135 |

Table S2. Model estimates: random intercept

| **Parameter** | **Estimate** | **P-value** | **Lower 95%CI** | **Upper 95%CI** |
| --- | --- | --- | --- | --- |
| Age: years | 1.016 | 0.000 | 1.015 | 1.018 |
| Age squared | 1.000 | 0.000 | 1.000 | 1.000 |
| Ventilation | 1.474 | 0.000 | 1.381 | 1.573 |
| APACHE III score: centered | 1.064 | 0.000 | 1.061 | 1.067 |
| APACHE III score squared | 1.000 | 0.000 | 1.000 | 1.000 |
| Ventilation x APACHE III score | 0.987 | 0.000 | 0.983 | 0.990 |
| Ventilation x APACHE III score squared | 1.000 | 0.001 | 1.000 | 1.000 |
| *Non-surgical diagnostic category*  Cardiovascular_medical | 1.000 |  | 1.000 | 1.000 |
| Respiratory medical | 1.306 | 0.000 | 1.180 | 1.445 |
| Liver_Gastro-intestinal_medical | 0.629 | 0.000 | 0.538 | 0.736 |
| Central nervous system: medical | 1.562 | 0.000 | 1.385 | 1.762 |
| Sepsis | 0.731 | 0.000 | 0.641 | 0.834 |
| Trauma | 0.565 | 0.000 | 0.475 | 0.673 |
| Metabolic Hormonal | 0.149 | 0.000 | 0.117 | 0.190 |
| Haematologic | 1.445 | 0.023 | 1.053 | 1.983 |
| Renal_Genito-urinary system | 0.472 | 0.000 | 0.367 | 0.608 |
| Other medical disorders | 0.610 | 0.005 | 0.432 | 0.859 |
| *Surgical diagnostic category*  Cardio-Vascular elective | 0.145 | 0.000 | 0.123 | 0.171 |
| Thoracic elective | 0.390 | 0.000 | 0.312 | 0.487 |
| Gastro-intestinal elective | 0.376 | 0.000 | 0.326 | 0.433 |
| Central nervous system elective | 0.435 | 0.000 | 0.351 | 0.539 |
| Traumatic/Orthopaedic elective | 0.199 | 0.000 | 0.155 | 0.256 |
| Renal_Genito-urinary system elective | 0.123 | 0.000 | 0.080 | 0.188 |
| Gynaecological/Oncological/Hormonal elective | 0.098 | 0.000 | 0.052 | 0.187 |
| Cardio-Vascular emergency | 0.446 | 0.000 | 0.357 | 0.558 |
| Thoracic emergency | 0.665 | 0.004 | 0.503 | 0.879 |
| Gastro-intestinal emergency | 0.518 | 0.000 | 0.454 | 0.592 |
| Central nervous system emergency | 1.652 | 0.000 | 1.396 | 1.956 |
| Traumatic/Orthopaedic emergency | 0.457 | 0.000 | 0.379 | 0.550 |
| Renal_Genito-urinary system emergency | 0.257 | 0.000 | 0.153 | 0.431 |
| Gynaecological/Oncological/Hormonal emergency | 0.154 | 0.000 | 0.078 | 0.303 |
| Cardiovascular_medical x APACHE III score | 1.000 |  | 1.000 | 1.000 |
| Respiratory medical x APACHE III score | 0.992 | 0.000 | 0.989 | 0.994 |
| Liver_Gastro-intestinal_medical x APACHE III score | 1.006 | 0.003 | 1.002 | 1.010 |
| Central nervous system: medical x APACHE III score | 0.997 | 0.115 | 0.994 | 1.001 |
| Sepsis x APACHE III score | 0.998 | 0.295 | 0.996 | 1.001 |
| Trauma x APACHE III score | 1.016 | 0.000 | 1.011 | 1.021 |
| Metabolic Hormonal x APACHE III score | 1.006 | 0.017 | 1.001 | 1.011 |
| Haematologic x APACHE III score | 0.993 | 0.108 | 0.985 | 1.001 |
| Renal_Genito-urinary system x APACHE III score | 0.994 | 0.054 | 0.988 | 1.000 |
| Other medical disorders x APACHE III score | 1.001 | 0.753 | 0.992 | 1.011 |
| Cardio-Vascular elective x APACHE III score | 1.007 | 0.015 | 1.001 | 1.012 |
| Thoracic elective x APACHE III score | 0.996 | 0.475 | 0.987 | 1.006 |
| Gastro-intestinal elective x APACHE III score | 0.995 | 0.049 | 0.990 | 1.000 |
| Central nervous system elective x APACHE III score | 0.994 | 0.260 | 0.984 | 1.004 |
| Traumatic/Orthopaedic elective x APACHE III score | 1.007 | 0.168 | 0.997 | 1.017 |
| Renal_Genito-urinary system elective x APACHE III score | 1.003 | 0.717 | 0.988 | 1.017 |
| Gynaecological/Oncological/Hormonal elective x APACHE III score | 1.020 | 0.158 | 0.992 | 1.049 |
| Cardio-Vascular emergency x APACHE III score | 1.003 | 0.287 | 0.998 | 1.008 |
| Thoracic emergency x APACHE III score | 0.995 | 0.287 | 0.986 | 1.004 |
| Gastro-intestinal emergency x APACHE III score | 0.997 | 0.109 | 0.994 | 1.001 |
| Central nervous system: emergency x APACHE III score | 0.995 | 0.078 | 0.990 | 1.001 |
| Traumatic/Orthopaedic emergency x APACHE III score | 1.011 | 0.000 | 1.006 | 1.017 |
| Renal_Genito-urinary system emergency x APACHE III score | 0.997 | 0.626 | 0.983 | 1.010 |
| Gynaecological/Oncological/Hormonal emergency x APACHE III score | 1.007 | 0.440 | 0.989 | 1.027 |
| No transfer to ICU | 1.000 |  | 1.000 | 1.000 |
| Inter-hospital transfer | 0.810 | 0.000 | 0.757 | 0.867 |
| Direct ICU-ICU transfer | 0.962 | 0.662 | 0.807 | 1.146 |
| Year 2010 | 0.952 | 0.018 | 0.913 | 0.991 |
| Cosine monthly | 0.969 | 0.736 | 0.805 | 1.166 |
| Sine monthly | 0.995 | 0.958 | 0.827 | 1.197 |
| Sine 6 monthly | 1.032 | 0.030 | 1.003 | 1.062 |
| Cosine 6 monthly | 1.013 | 0.372 | 0.985 | 1.043 |
| Sine weekly | 1.029 | 0.760 | 0.856 | 1.237 |
| Cosine weekly | 0.995 | 0.962 | 0.827 | 1.199 |
| Constant | 0.075 | 0.000 | 0.067 | 0.084 |
| Random effects: ICU site (identity) standard deviation | 0.303 | 0.000 | 0.257 | 0.358 |
| Intraclass correlation (ICU site) | 0.027 |  | 0.020 | 0.038 |

Table S3. Model estimates: random coefficient

| **Parameter** | **Estimate** | **P-value** | **Lower 95%CI** | **Upper 95%CI** |
| --- | --- | --- | --- | --- |
| Age: years | 1.017 | 0.000 | 1.015 | 1.018 |
| Age squared | 1.000 | 0.000 | 1.000 | 1.000 |
| Ventilation | 1.476 | 0.000 | 1.382 | 1.577 |
| APACHE III score: centered | 1.064 | 0.000 | 1.060 | 1.067 |
| APACHE III score squared | 1.000 | 0.000 | 1.000 | 1.000 |
| Ventilation x APACHE III score | 0.987 | 0.000 | 0.983 | 0.990 |
| Ventilation x APACHE III score squared | 1.000 | 0.002 | 1.000 | 1.000 |
| *Non-surgical diagnostic category*  Cardiovascular_medical | 1.000 |  | 1.000 | 1.000 |
| Respiratory medical | 1.295 | 0.000 | 1.170 | 1.433 |
| Liver_Gastro-intestinal_medical | 0.625 | 0.000 | 0.535 | 0.731 |
| Central nervous system: medical | 1.559 | 0.000 | 1.381 | 1.759 |
| Sepsis | 0.729 | 0.000 | 0.639 | 0.833 |
| Trauma | 0.557 | 0.000 | 0.468 | 0.664 |
| Metabolic Hormonal | 0.148 | 0.000 | 0.116 | 0.188 |
| Haematologic | 1.440 | 0.024 | 1.049 | 1.976 |
| Renal_Genito-urinary system | 0.475 | 0.000 | 0.369 | 0.613 |
| Other medical disorders | 0.597 | 0.003 | 0.424 | 0.842 |
| *Surgical diagnostic category*  Cardio-Vascular elective | 0.146 | 0.000 | 0.124 | 0.173 |
| Thoracic elective | 0.395 | 0.000 | 0.316 | 0.494 |
| Gastro-intestinal elective | 0.379 | 0.000 | 0.328 | 0.437 |
| Central nervous system elective | 0.444 | 0.000 | 0.358 | 0.551 |
| Traumatic/Orthopaedic elective | 0.202 | 0.000 | 0.157 | 0.260 |
| Renal_Genito-urinary system elective | 0.125 | 0.000 | 0.081 | 0.192 |
| Gynaecological/Oncological/Hormonal elective | 0.102 | 0.000 | 0.054 | 0.194 |
| Cardio-Vascular emergency | 0.441 | 0.000 | 0.353 | 0.552 |
| Thoracic emergency | 0.672 | 0.005 | 0.508 | 0.889 |
| Gastro-intestinal emergency | 0.511 | 0.000 | 0.447 | 0.584 |
| Central nervous system: emergency | 1.656 | 0.000 | 1.397 | 1.963 |
| Traumatic/Orthopaedic emergency | 0.455 | 0.000 | 0.378 | 0.547 |
| Renal_Genito-urinary system emergency | 0.258 | 0.000 | 0.154 | 0.432 |
| Gynaecological/Oncological/Hormonal emergency | 0.153 | 0.000 | 0.078 | 0.302 |
| Cardiovascular_medical x APACHE III score | 1.000 |  | 1.000 | 1.000 |
| Respiratory medical x APACHE III score | 0.992 | 0.000 | 0.989 | 0.994 |
| Liver_Gastro-intestinal_medical x APACHE III score | 1.006 | 0.002 | 1.002 | 1.010 |
| Central nervous system: medical x APACHE III score | 0.997 | 0.123 | 0.994 | 1.001 |
| Sepsis x APACHE III score | 0.998 | 0.260 | 0.996 | 1.001 |
| Trauma x APACHE III score | 1.016 | 0.000 | 1.011 | 1.021 |
| Metabolic Hormonal x APACHE III score | 1.006 | 0.017 | 1.001 | 1.011 |
| Haematologic x APACHE III score | 0.993 | 0.117 | 0.985 | 1.002 |
| Renal_Genito-urinary system x APACHE III score | 0.994 | 0.045 | 0.988 | 1.000 |
| Other medical disorders x APACHE III score | 1.002 | 0.635 | 0.993 | 1.012 |
| Cardio-Vascular elective x APACHE III score | 1.007 | 0.016 | 1.001 | 1.012 |
| Thoracic elective x APACHE III score | 0.997 | 0.566 | 0.987 | 1.007 |
| Gastro-intestinal elective x APACHE III score | 0.996 | 0.125 | 0.991 | 1.001 |
| Central nervous system: elective x APACHE III score | 0.995 | 0.290 | 0.985 | 1.005 |
| Traumatic/Orthopaedic elective x APACHE III score | 1.007 | 0.151 | 0.997 | 1.017 |
| Renal_Genito-urinary system elective x APACHE III score | 1.004 | 0.601 | 0.989 | 1.019 |
| Gynaecological/Oncological/Hormonal elective x APACHE III score | 1.021 | 0.163 | 0.992 | 1.050 |
| Cardio-Vascular emergency x APACHE III score | 1.003 | 0.259 | 0.998 | 1.009 |
| Thoracic emergency x APACHE III score | 0.995 | 0.287 | 0.986 | 1.004 |
| Gastro-intestinal emergency x APACHE III score | 0.998 | 0.186 | 0.995 | 1.001 |
| Central nervous system: emergency x APACHE III score | 0.995 | 0.085 | 0.990 | 1.001 |
| Traumatic/Orthopaedic emergency x APACHE III score | 1.011 | 0.000 | 1.006 | 1.017 |
| Renal_Genito-urinary system emergency x APACHE III score | 0.997 | 0.616 | 0.983 | 1.010 |
| Gynaecological/Oncological/Hormonal emergency x APACHE III score | 1.008 | 0.423 | 0.989 | 1.027 |
| No transfer to ICU | 1.000 |  | 1.000 | 1.000 |
| Inter-hospital transfer | 0.810 | 0.000 | 0.757 | 0.867 |
| Direct ICU-ICU transfer | 0.952 | 0.585 | 0.798 | 1.136 |
| Year 2010 | 0.949 | 0.013 | 0.911 | 0.989 |
| *Locality*  Northern Territory | 1.000 |  | 1.000 | 1.000 |
| New South Wales | 1.081 | 0.726 | 0.699 | 1.674 |
| Australian Capital Territory | 1.045 | 0.883 | 0.582 | 1.876 |
| South Australia | 0.985 | 0.950 | 0.606 | 1.601 |
| Victoria | 1.046 | 0.840 | 0.674 | 1.625 |
| Western Australia | 0.970 | 0.921 | 0.530 | 1.776 |
| New Zealand | 1.275 | 0.312 | 0.796 | 2.040 |
| Queensland | 1.025 | 0.914 | 0.659 | 1.593 |
| Tasmania | 1.303 | 0.341 | 0.756 | 2.246 |
| *Hospital descriptor*  Rural | 1.000 |  | 1.000 | 1.000 |
| Metropolitan | 1.039 | 0.629 | 0.889 | 1.214 |
| Tertiary | 1.025 | 0.820 | 0.830 | 1.265 |
| Private | 0.767 | 0.002 | 0.648 | 0.908 |
| Mean site volume (centred) | 1.000 | 0.597 | 1.000 | 1.000 |
| Cosine monthly | 0.970 | 0.751 | 0.806 | 1.168 |
| Sine monthly | 0.992 | 0.932 | 0.824 | 1.194 |
| Sine 6 monthly | 1.033 | 0.026 | 1.004 | 1.063 |
| Cosine 6 monthly | 1.013 | 0.380 | 0.984 | 1.042 |
| Sine weekly | 1.033 | 0.728 | 0.859 | 1.243 |
| Cosine weekly | 0.993 | 0.943 | 0.825 | 1.196 |
| Constant | 0.075 | 0.000 | 0.048 | 0.118 |
| Random effects: APACHE III score (standard deviation) | 0.005 | 0.000 | 0.003 | 0.006 |
| Random effects: ICU site (standard deviation) | 0.245 | 0.000 | 0.198 | 0.304 |
| Random effects: correlation (APACHE III, ICU site) | 0.005 | 0.981 | -0.382 | 0.391 |
| Intraclass correlation (ICU site) | 0.020 |  | 0.118 | 0.027 |

Figure S1. Standardized normal probability plots (P–P plot) of the random effects; random intercept model

Figure S2. Standardized normal probability plots (P–P plot) of the random effects; random coefficient model

1. Random effects for ICU site: APACHE III score

1. Random effects for ICU site: constant
